# Risk behaviours and non-atopic comorbidities of adolescents with asthma^☆^

**DOI:** 10.1016/j.waojou.2025.101093

**Published:** 2025-07-17

**Authors:** Trine Mølbæk-Engbjerg, Nilo Vahman, Mina Ali, Frederikke Skov, Rebecca Vinding, David Horner, Nicklas Brustad, Jonathan Thorsen, Ann-Marie M. Schoos, Jakob Stokholm, Klaus Bønnelykke, Bo Chawes

**Affiliations:** a COPSAC, Copenhagen Prospective Studies on Asthma in Childhood, Herlev and Gentofte Hospital, University of Copenhagen, Copenhagen, Denmark; b Department of Paediatrics, Slagelse Hospital, Slagelse, Denmark; c Department of Clinical Medicine, Faculty of Health and Medical Sciences, University of Copenhagen, Copenhagen, Denmark; d Department of Food Science, University of Copenhagen, Frederiksberg C, Denmark

**Keywords:** Asthma, Obesity, Neuropsychiatric diagnosis, Self-destructive behaviour, Screen time

## Abstract

**Background:**

Risk behaviours, obesity, and neuropsychiatric comorbidity have been demonstrated in various chronic diseases but are less well described among adolescents with asthma.

**Methods:**

We clinically assessed asthma status at the 18-year follow-up visit of the Danish Copenhagen Prospective Studies on Asthma in Childhood (COPSAC_2000_) birth cohort born to mothers with asthma, and we investigated risk behaviours and non-atopic comorbidities. We included obesity and neuropsychiatric diseases captured from medical records and electronic questionnaires on behavioural traits and psychopathology. Associations between asthma status, risk behaviours, and non-atopic comorbidity were analysed using logistic regression models.

**Results:**

A total of 370 individuals (90%) completed the 18-year visit, and 93 of these (25.1%) had current asthma. Comparing adolescents with and without asthma, binge drinking was reported in 75.2% vs 66.0%, current smoking in 26.9% vs 32.9%, and drug use in 16.1% vs 26.0%. High daily screen use was reported in 25.8% vs 16.6%; 26.9% vs 17.3% reported self-destructive behaviour; 24.7% vs 14.1% had neuropsychiatric comorbidity, and 10.8% vs 6.9% had obesity. In univariate analyses, asthma was associated with self-destructive behaviour, OR = 1.84 (1.03–3.21), p = 0.035, and neuropsychiatric comorbidity, OR = 2.01 (1.11–3.56), p = 0.019. In multivariable analysis with backward selection, asthma was associated with neuropsychiatric comorbidity, OR = 2.04 (1.004–4.12), p = 0.049, a trend of increased self-destructive behaviour, OR = 1.76 (0.93–3.29), p = 0.079, and less drug use, OR = 0.59 (0.29–0.96), p = 0.045.

**Conclusion:**

Asthma was associated with neuropsychiatric comorbidity and self-destructive behaviour, but less drug use. There were no consistent associations with other risk behaviours or obesity.

**Key message**

Risk behaviours, obesity, and neuropsychiatric comorbidity have been demonstrated in chronic diseases but are less well described among adolescents with asthma. In our cohort at risk of asthma, adolescents with asthma had a higher risk of neuropsychiatric comorbidity and a trend of more self-destructive behaviour. These findings underscore that adolescents with asthma have an increased risk of chronic non-atopic disease and self-harm, which clinicians should be aware of in the regular monitoring and treatment of asthma.

## Introduction

Risk behaviours are conscious actions that increase the risk of harmful health consequences, a well-known adolescent phenomenon, to promote independence and help establish autonomy.^1^ Existing literature shows that young adults living with a chronic disease behave in a more risk-taking manner than their healthy peers.^2^^,^^3^ An American study, for example, has shown that adolescents with asthma were more likely to report cigarette smoking, drug use, and binge drinking as compared to their healthy peers.^3^

Adolescents with asthma also have an increased risk of developing depression and anxiety compared to adolescents without asthma,^4^ and more recently, a link between asthma and neurodevelopmental disorders, such as attention deficit hyperactivity disorder (ADHD), has been shown.^5^ Asthma has also been associated with dyslipidemia,^6^^,^^7^ and it is hypothesised that obesity and asthma are associated because of shared genetics, environmental risk factors, and underlying low-grade systemic inflammation.^8^

Using descriptive analysis, this study aims to investigate risk behaviours, obesity, and neuropsychiatric comorbidity in a well-characterised cohort of 18-year-olds with and without asthma.

## Methods

### Study population

The COPSAC_2000_ cohort is a single-centre, prospective birth cohort study consisting of 411 children born to mothers with asthma. The children were included at 1 month of age and visited the COPSAC research unit at scheduled visits every 6 months until age 7 and again at ages 12 and 18. Furthermore, the children visited the research unit upon the occurrence of any acute airway or skin symptoms. Recruitment and baseline characteristics have previously been described in detail.^9^

### Risk behaviours

At the 18-year follow-up visit, the participants were interviewed about their risk behaviours, including alcohol consumption, binge drinking, tobacco smoking, drug use, screen time use, and self-destructive behaviour. The participants were interviewed without their parents being present.

#### Alcohol consumption

The participants were asked if they had ever consumed more than 1 unit of alcohol and at what age this first occurred. They were then asked about their alcohol consumption in the past year, including frequency of drinking (never, max once per month, 2–4 times per month, 2–3 times per week, or ≥ 4 times per week), and numbers of units per occasion (0 units, 1–2 units, 3–4 units, 5–6 units, 7–9 units or ≥ 10 units). Finally, they were asked about the number of episodes in which they consumed 5 or more units of alcohol on a single occasion, such as binge drinking.

#### Tobacco smoking

The participants were interviewed about the age at which they first smoked a cigarette, their current smoking status, frequency of smoking (daily, weekly, less than weekly, or never), and estimated daily cigarette consumption over the past 4 weeks. Additionally, they were asked if they had experience with e-cigarettes. Current smoking was defined as smoking within the last week, including daily smoking, and occasional smoking was defined as smoking less than weekly but within the previous 4 weeks.

#### Drug use

The participants were asked about their prior experience with drug use, answering an online questionnaire (part of the Depression, Anxiety, and Stress Scale [DASS21]), and, if applicable, which substances they had tried, including cannabinoids, amphetamine, ecstasy/3,4-methylenedioxy-N-methylamphetamine (MDMA)/fantasy, cocaine, opiates (such as heroin, morphine, or methadone), Lysergic Acid Diethylamide (LSD), sedatives, inhalation of lighter gas, inhalation of nitrous oxide (“laughing gas”), hallucinogenic mushrooms, or others. They were then asked to specify the frequency of their drug use, which was categorised as never, once, less than once per month, monthly, weekly, daily, or almost daily.

#### Self-destructive behaviour

The frequency of self-destructive behaviours including cutting, scratching, hitting, and overeating, was captured as part of the DASS21 online questionnaire. The participants were asked whether they had ever done self-destructive behaviour (yes/no). If they answered yes, they were asked how often they had done that in the previous year and they were given the possibility to specify the type of self-destructive behaviour. After completing the online questionnaire, the participants returned to the research clinic staff. If any concerns or questions were raised as a result of the questionnaire, they were addressed directly by the staff. In cases where further support was needed, the participants and/or their parents were guided on how to access appropriate services within the Danish healthcare system. In the analyses, we used any self-destructive behaviour (yes/no).

#### Screen time

The participants were interviewed about their screen time habits over the past year, distinguishing between screen time on a television (TV) screen, a telephone, tablet, or computer. Screen time was categorised based on use during weekdays (Monday to Thursday) and weekends (Friday to Sunday) as the average number of hours spent on screen during leisure time. The total screen time was taken as an addition of TV screen, telephone, tablet, or computer. For this study, weekday and weekend screen time were combined. High daily screen time use was defined as exceeding 8 h per day.

### Lung function tests and asthma diagnosis

Lung function was measured by spirometry (MasterScope Pneumoscreen, Erich Jäeger, Würzburg, Germany),^10^ and specific airway resistance (sRaw) was assessed by whole-body plethysmography (MasterScope Bodybox, Erich Jäeger, Würzburg, Germany).^11^ Fractional exhaled nitric oxide (FeNO) was measured using the Denox 88 version 88 DX0256, Switzerland.^12^

Asthma was solely diagnosed by the COPSAC physicians.^13^ Based on the European Respiratory Society (ERS) recommendations,^14^ an asthma diagnosis was confirmed if at least 2 of the following criteria were fulfilled: 1) Obstructive lung function (FEV1<80% and/or FEV_1_/FVC<80% and/or sRaw>1.6 kPa/s); 2) Bronchodilator reversibility (change of FEV1≥12% and/or sRaw≥30% from baseline after inhalation of short-acting beta-2-agonist); 3) Airway inflammation (FeNO≥25 ppb); and/or 4) Bronchial hyperresponsiveness as measured by methacholine challenge test (drop in FEV1≥20% from baseline by a dose of methacholine ≤8 mg/ml) and/or exercise test (≥10% decrease in FEV1 from baseline). Remission was defined as no symptoms or asthma treatment for 1 year.

### Non-atopic comorbidity

#### Neuropsychiatric disorders

The diagnoses were obtained from the medical records based on ICD-10 criteria, including any neuropsychiatric diagnosis, former and present. The diagnoses were grouped into the following groups: ADHD, autism spectrum disorder, anxiety, depression, eating disorder, mental retardation, personality disorder, schizophrenia, post-traumatic stress disorder (PTSD), obsessive-compulsive disorder (OCD), and others (Supplemental Appendix for details).

During the 18-year visit, 3 electronic questionnaires on behavioural traits and psychopathology were completed: 1. The strengths and difficulties questionnaire (SDQ),^15^ 2. The Adult ADHD Self-Report Scale (ASRS),^16^ and 3. The DASS21^17^ (Supplemental Appendix for details).

#### BMI and obesity

Weight was measured under standard conditions without clothing but in underwear, using a calibrated digital scale. Height was measured by a Harpenden stadiometer (Holtain Ltd, Crymych, Dyfed, Wales) as previously described.^9^ Body mass index (BMI) was calculated as weight in kilograms divided by height in meters squared and grouped according to the World Health Organisation (WHO) definition as underweight: BMI<18.5; normal weight: 18.5–25; overweight: 25–30, and obesity: >30.^18^

### Statistical analysis

The associations between asthma and risk behaviours, obesity, and neuropsychiatric disorders were analysed using logistic regression models. Data were analysed in a univariate- and multivariable regression model adjusted for sex, gestational age (GA), mother's age, mother's education, household income, breastfeeding, and smoking during pregnancy. The multivariable model also included all risk behaviours and non-atopic comorbidities: binge drinking, current and occasional smoking, drug use, self-destructive behaviour, high daily screen time use, neuropsychiatric disorder, and obesity. To simplify the model and clarify which variables explain the most, a backward selection procedure was applied by successively dropping the parameter with the highest p-value, keeping only variables with p < 0.10 in the final model. Finally, interaction with sex was explored, and post hoc analyses were stratified for sex. Data processing was conducted with R (R Foundation for Statistical Computing, R version 4.0.3 (2020-10-10), Vienna, Austria). Due to the exploratory nature of the study, we did not adjust for multiple comparisons.

### Ethics

The study was conducted following the guiding principles of the Declaration of Helsinki. It was approved by the Local Ethics Committee (COPSAC_2000_: H–B-2008-093, COPSAC_2000_ 18- & 24-years follow-up: H-16039498) and the Danish Data Protection Agency (COPSAC_2000_: 2015-41-3696). Both parents gave written informed consent before enrolment. At the COPSAC_2000_ 18-year visit, the study participants also gave written consent.

## Results

### Demographics

At the 18-year visit, follow-up was completed for 370 (90%) of the 411 participants in the cohort, where 93 participants (25.1%) had concurrent asthma, and (44.3%) had an asthma diagnosis at any time earlier in childhood. Demographics are detailed in Table 1, showing that among adolescents with vs without asthma there was an increased prevalence of allergic rhinitis (66.7% vs 29.2%, p < 0.001) and more days with passive tobacco smoke exposure (p = 0.03).

**Table 1:** Demographic information of the probands included in the COPSAC_2000_ cohort at the 18-year visit.

Demographics, N (%)	All N (%) N = 370	Current asthma N = 93	Not current asthma N = 277	p-value
Allergic rhinitis, current	143 (38.6)	62 (66.7)	81 (29.2)	**<0.001**
Atopic dermatitis, current	38 (10.3)	15 (16.1)	23 (8.3)	0.19
Parents' divorce	142 (38.4)	38 (40.9)	104 (37.5)	0.66
Race (Caucasian)	355 (96.7)	89 (95.7)	266 (96.0)	1.00
Fireplace				0.92
1–100 days	137 (37.0)	36 (38.7)	101 (36.5)	
>100 days	38 (10.3)	9 (9.7)	29 (10.5)	
Furred animals				
Cats, yes	98 (26.5)	21 (22.6)	77 (27.8)	0.40
Dogs, yes	179 (48.4)	43 (46.2)	136 (49.1)	0.74
Passive smoking				**0.03**
1–100 days per years	116 (31.4)	36 (38.7)	80 (28.9)	
>100 days per year	110 (29.7)	18 (19.4)	92 (33.2)	
Other children younger, yes	217 (58.6)	46 (49.5)	171 (61.7)	0.12
Other children older, yes	87 (23.5)	26 (28.0)	61 (22.0)	0.45
Proband education				0.38
Elementary school, completed	357 (96.5)	91 (97.8)	266 (96.0)	
College, started	288 (77.8)	72 (77.4)	216 (78.0)	
Tradesman, started	38 (10.3)	5 (7.94)	33 (12.0)	
University, started	1 (0.3)	1 (1.1)	–	
Parents education, completed				0.33
Elementary school, mother	13 (3.5)	5 (5.4)	6 (2.2)	
Elementary school, father	17 (4.6)	4 (4.3)	13 (4.7)	
High school, mother	6 (1.6)	1 (1.1)	5 (1.8)	
High school, father	7 (1.9)	4 (4.3)	3 (1.1)	
Tradesman, mother	94 (25.4)	33 (35.5)	61 (22.0)	
Tradesman, father	102 (27.6)	23 (24.7)	79 (28.5)	
University, medium long, mother	125 (33.8)	29 (31.2)	96 (34.7)	
University, medium long, father	70 (18.9)	12 (12.9)	58 (20.9)	
University, long, mother	69 (18.6)	16 (17.2)	53 (19.1)	
University, long, father	82 (22.2)	25 (26.9)	57 (20.6)	

Bold indicates p-values < 0.05.

The table presents demographic information of the probands included in the COPSAC_2000_ cohort at the 18-year visit

### Risk behaviours

As shown in Supplemental Fig. 1, certain risk behaviours were inter-correlated, such as smoking and drug use (r = 0.52) and alcohol use (r = 0.21) and self-esteem and smoking (r = −0.18), drug (r = −0.16) and alcohol use (r = −0.21), which showed the strongest positive and negative correlations, respectively.

#### Alcohol consumption

Among adolescents with asthma, 64.5% reported drinking more than once per month, and when drinking, 75.2% drank ≥5 units per occasion. Of those who drank ≥5 units per event, 20.4% drank >10 units of alcohol per event. Among adolescents without asthma, 69.7% reported drinking more than once per month, and when drinking, 66.0% drank ≥5 units per event. Of those adolescents without asthma who drank ≥5 units per event, 22.7% drank >10 units (Table 2). No significant associations existed between asthma status and binge drinking in the univariate- or multivariable models (Table 3).

**Table 2:** Descriptive information on risk behaviours from probands in the cohort.

Risk behaviours	All N = 370	Current asthma N = 93	Not current asthma N = 277	p-value^c^
Alcohol; drinking frequency, N = 342 (92.4)				0.46
Never	3 (1.0)	–	3 (1.0)	
Max once per month	89 (24.1)	26 (28.0)	61 (22.0)	
>1 per month	253 (68.4)	60 (64.5)	193 (69.7)	
Alcohol; units				0.28
1–4	88 (23.8)	16 (17.2)	71 (25.6)	
5–9	172 (46.5)	51 (54.8)	120 (43.3)	
>10	82 (22.2)	19 (20.4)	63 (22.7)	
Smoking, N = 189 (51.1)	116 (31.4)	25 (26.9)	91 (32.9)	0.34
Current smoking^a^	65 (17.6)	16 (17.2)	49 (17.7)	
Occasional smoking^a^	51 (13.8)	9 (9.7)	42 (15.2)	
Former smoker	73 (19.7)	18 (19.4)	55 (19.9)	
Daily	28 (7.6)	5 (5.4)	23 (8.3)	0.35
1-4 cigarettes per day	11 (39.3)	3 (60.0)	8 (34.7)	
5-9 cigarettes per day	8 (28.6)	2 (40.0)	6 (26.1)	
>9 cigarettes per day	9 (32.1)	–	9 (39.1)	
Drug use ever	87 (23.8)	15 (16.1)	72 (26.0)	**<0.01**
Cannabinoids	60 (70.0)	9 (60.0)	51 (70.8)	
Cocaine	22 (25.3)	5 (33.3)	17 (23.6)	
Laughing gas (NO)	18 (20.7)	1 (6.7)	17 (23.6)	
Ecstasy/MDMA/Fantasy	12 (13.9)	4 (26.7)	8 (11.1)	
Others^b^	34 (39.1)	7 (46.7)	27 (37.5)	
Screen time, N = 367 (99.2)				
Total screen time hours, median (IQR)	6.0 (4.5–7.0)	6.0 (4.5–8.0)	6.0 (4.5–7.0)	0.93
High daily screen time use	70 (18.9)	24 (25.8)	46 (16.6)	**0.01**
Self-destructive behaviour	73 (19.7)	25 (26.9)	48 (17.3)	**<0.01**

Bold indicates p-values < 0.05.

The table presents descriptive information on risk behaviour from probands in the cohort. The information is presented in groups of asthma yes/no.

a Current smoking was defined as daily or weekly smoking, and occasional smoking was defined as smoking less than weekly.

b Etc. Amphetamine, sedatives, LSD, opiates, euphoric mushrooms, lighter gas.

c Significance between the genders is calculated with a *t*-test. In cases of not normal distributed data Wilcox test has been used

**Table 3:** Association between risk behaviours, obesity, and neuropsychiatric disorders.

Asthma associations		Univariate model		Fully adjusted model			Backward selection model		
*Predictors*	*OR*	*CI*	*p*	*OR*	*CI*	*p*	*OR*	*CI*	*p*
Binge drinking^a^	1.66	(0.93–3.08)	0.10	1.70	(0.88–3.43)	0.12			
Current smoking^b^	0.86	(0.44–1.62)	0.64	0.50	(0.17–1.29)	0.17	–	–	–
Occasional smoking^b^	0.56	(0.24–1.19)	0.15	0.55	(0.21–1.33)	0.20	–	–	–
Drug use (yes)	0.57	(0.30–1.05)	0.081	0.73	(0.32–1.62)	0.44	0.59	(0.29–0.96)	**0.045**
High daily screen time use^c^	1.74	(0.98–3.04)	0.053	1.68	(0.83–3.35)	0.15	–	–	–
Self-destructive behaviour (yes)	1.84	(1.03–3.21)	**0.035**	1.65	(0.81–3.30)	0.16	1.76	(0.93–3.29)	0.079
Neuropsychiatric disorder^d^	2.01	(1.11–3.56)	**0.019**	2.17	(1.04–4.48)	**0.037**	2.04	(1.004–4.12)	**0.049**
Obesity	1.55	(0.66–3.44)	0.30	1.67	(0.61–4.36)	0.30	–	–	–

Bold indicates p-values < 0.05.

The adjusted model is adjusted for sex, GA, mother's age, educational level of the mother, household income, smoking during pregnancy, and breastfeeding. All risk behaviours are included in the multivariable model: binge drinking, smoking, drug use, high daily screen time use, self-destructive behaviour, neuropsychiatric disorder, and obesity.

a Binge drinking refers to alcohol units ≥5.

b Current smoking refers to smoking daily or weekly, and occasional smoking refers to smoking less than weekly.

c High daily screen time use >8 h/day.

d Neuropsychiatric disorder ever through childhood

#### Tobacco smoking

**Fig. 1:**
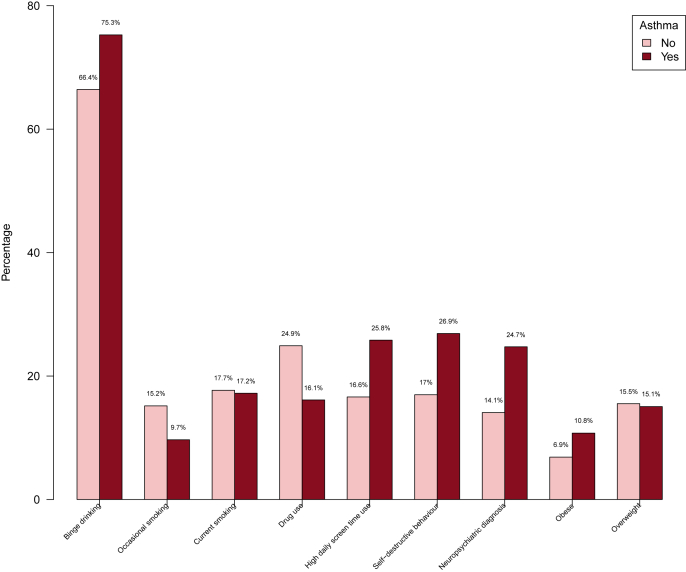
Distribution of risk behaviours, BMI, and neuropsychiatric disorders in adolescents with vs without asthma

At age 18 years, 26.9% of the adolescents with asthma were smokers (daily, weekly, or occasionally) vs 32.9% of adolescents without asthma (Table 2, Fig. 1). There was no significant association between tobacco smoking and asthma status (Table 3).

#### Drug use

Experience with any drug use tended to be less frequent among adolescents with vs without asthma in the univariate analysis: 16.1% vs 26.0%, OR = 0.57 (95% CI, 0.30–1.05), p = 0.08 (Table 3, Fig. 1). Among adolescents with vs without asthma, 60.0% vs 70.8% had tried cannabinoids, 33.3% vs 23.6% had tried cocaine, 6.7% vs 23.6% had inhaled laughing gas, and 26.7% vs 11.1% had tried MDMA. After the backward selection procedure, the multivariable model showed a significant inverse association between lower drug use and risk of asthma: adjusted OR = 0.59 (0.29–0.96), p = 0.045 (Table 3, Fig. 1).

#### Screen time

The median screen time per day was 6.0 h in both adolescents with and without asthma, whereas there was a trend of more adolescents with vs without asthma who had a high daily screen time use, ie, screen time >8h/day: 25.8% vs 16.6%, OR = 1.74 (0.98–3.04), p = 0.053 (Table 2). However, this association was not significant in the multivariable and backward selection models (Table 3).

#### Self-destructive behaviour

Self-destructive behaviour was reported in 26.9% of adolescents with asthma compared to 17.3% of adolescents without asthma (Table 2). In the univariate analysis, asthma was associated with significantly more self-destructive behaviour, OR = 1.84 (1.03–3.21), p = 0.035, but the multivariable model after backward selection only showed a trend of increased risk among adolescents with asthma, OR = 1.76 (0.93–3.29), p = 0.08 (Table 3).

### Non-atopic comorbidity

#### Neuropsychiatric disorders

A total of 16.8% of the participants had been diagnosed with a neuropsychiatric diagnosis at some point in childhood. The distribution of specific diagnosis among children with any diagnosis is visualised in Supplemental Fig. 2. Adolescents with vs without asthma had a doubled risk of neuropsychiatric comorbidity, 24.7% vs 14.1%, OR = 2.01 (1.11–3.56), p = 0.019, which was confirmed in the multivariable analysis, OR = 2.17 (1.04–4.48), p = 0.037, and in backward selection procedure OR = 2.04 (1.004–4.12), p = 0.049 (Table 3).

The most frequent neuropsychiatric diagnosis was ADHD, which was diagnosed in 10.8% of adolescents with asthma compared to 5.8% without asthma. Autism was diagnosed in 7.5% of adolescents with asthma vs 1.8% without asthma, depression in 4.3% vs 1.2%, eating disorders in 3.2% vs 2.9%, OCD in 3.2% vs 2.2%, and anxiety in 2.2% vs 3.6%. There was a significant difference in autism between adolescents with vs without asthma (p = 0.01) but not for the other specific neuropsychiatric diagnoses (Table 4).

**Table 4:** Association between BMI categories, specific neuropsychiatric disorders, and asthma.

BMI and neuropsychiatric disorders, N (%)	All N = 370	Current asthma N = 93	Not current asthma N = 277	p-value^d^
BMI, N = 367 (99.1)				0.58
BMI <18.5	32 (8.7)	6 (6.45)	26 (9.39)	
BMI 18.5–24.9	249 (67.8)	64 (68.8)	185 (66.8)	
BMI 25-30	57 (15.5)	14 (15.1)	43 (15.5)	
BMI >30	29 (7.90)	10 (10.8)	19 (6.9)	
ASRS questionnaire, N = 348 (94.0)^a^	4.0 (2.0–7.0)	4.5 (2.0–7.0)	4.0 (2.0–7.0)	0.97
SDQ - total difficulty score, N = 347 (93.7)^b^	74 (21.3)	22 (25.0)	52 (20.1)	0.59
SDQ - daily functioning score (impact score)^b^	95 (27.4)	23 (26.1)	72 (27.8)	0.94
Neuropsychiatric disorder^c^	62 (16.8)	23 (24.7)	39 (14.1)	**0.02**
ADHD	26 (7.0)	10 (10.8)	16 (5.8)	0.16
Depression	8 (2.2)	4 (4.3)	4 (1.4)	0.11
Anxiety	12 (3.2)	2 (2.2)	10 (3.6)	0.74
Autism	13 (3.1)	7 (7.5)	5 (1.8)	**0.01**
Eating disorder	11 (3.0)	3 (3.2)	8 (2.9)	1.00
Personality disorder	2 (0.5)	–	2 (0.7)	–
Mental retardation	3 (0.8)	1 (1.1)	2 (0.7)	1.00
PTSD	5 (1.4)	2 (2.2)	3 (1.1)	0.60
Schizophrenia	2 (0.5)	1 (1.1)	1 (0.4)	0.44
OCD	9 (2.4)	3 (3.2)	6 (2.2)	0.70

Bold indicates p-values < 0.05.

a ASRS (Adult ADHD self reported scale), data from 348 proband, 88 with asthma and 260 without asthmatic disease.

b SDQ-questionnaire. Data available from 347 probands.

c Neuropsychiatric disorders include a group of other diagnoses. This group includes 7 individuals. Some of the probands have more than 1 neuropsychiatric diagnosis, which is why the number of specific categories does not sum to 62.

d Fishers test

Data from the ASRS questionnaire were available from 348 children. Of those, the total score median (IQR) was 4 (2.0–7.0) in adolescents with asthma vs 4.5 (2.0–7.0) in adolescents without asthma (p = 0.97) (Table 4).

Data from the SDQ questionnaire were available from 347 children. Looking into the total difficulty score, 74 (21.3%) adolescents meet the criteria for an abnormal score. Of those, 22 (25.0%) were adolescents with asthma vs 52 (20.1%) without asthma, p = 0.59. In the impact score, 95 (27.4%) fulfilled the criteria for an abnormal score, and of those, 23 (26.1%) were adolescents with asthma vs 72 (27.8%) without asthma, p = 0.94 (Table 4).

#### Obesity

**Fig. 2:**
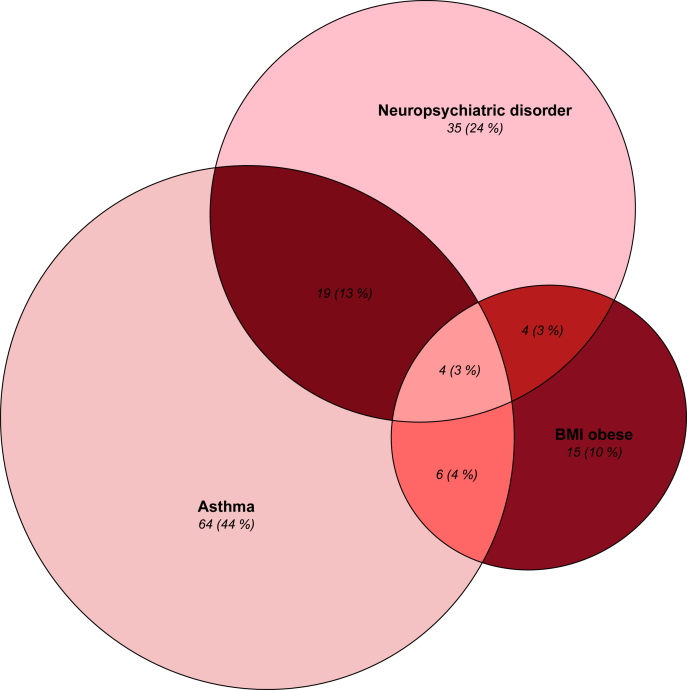
Venn diagram showing the relationship between the 147 adolescents with either asthma and/or neuropsychiatric disorders and/or BMI ≥30. The number in each category and the percentage of the 147 children is given in the figure as N (%)

Among adolescents with asthma, 15.1% were categorised as having overweight (BMI 25.1–30.0 kg/m^2^) and 10.8% as having obesity (BMI>30 kg/m^2^), which was 15.5% and 6.9% among adolescents without asthma (Table 4). No significant associations existed between BMI categories and asthma status (Table 3, Fig. 2).

### Post hoc analysis of gender differences

Asthma was associated with obesity among females, OR = 3.48 (1.18–10.33), p = 0.02, but not among males, OR = 1.55 (0.66–3.44), p = 0.30, which showed a significant gender difference (p-interaction = 0.04) (Supplemental Table 1). In stratified analyses, it appeared that asthma was more strongly associated with self-destructive behaviour among females, OR = 2.24 (1.10–4.56, p = 0.03), than males, OR = 1.31 (0.39–3.79), p = 0.64. However, there were no significant gender differences in the association between asthma and self-destructive behaviour or any other risk behaviours or neuropsychiatric comorbidity (p-interactions>0.05) (Supplemental Table 1).

## Discussion

### Primary findings

In our high-risk asthma cohort, we found that adolescents with asthma had a higher risk of neuropsychiatric comorbidity and a trend of a higher risk of self-destructive behaviour. In contrast, the risk of drug use was lower compared to adolescents without asthma. Other risk behaviours, such as tobacco smoking and alcohol consumption, were not associated with asthma, whereas obesity was only associated with asthma among females.

### Interpretation

The previously observed associations between risk behaviours and chronic diseases^1^ have been suggested to arise due to the following: First, adolescents living with a chronic disease have a higher need for acceptance from other adolescents. Second, parents of adolescents who live with a chronic disease may be less restrictive regarding rules and boundaries that do not directly involve the chronic disease. Third, chronic disease can cause less focus on risk behaviours and reduce preventive counselling.^1^

Genetic predisposition increases the risk of developing asthma,^19^ which concurs with the high number of participants with asthma in our cohort, where one-fourth had asthma compared to around 15% in the background population.^9^ In recent years, there has been an increased focus on the relationship between asthma and other non-communicable, lifestyle-related disorders such as obesity^20^, ^21^, ^22^ and neuropsychiatric disorders.^8^^,^^19^ We observed a prevalence of current or past neuropsychiatric comorbidity of 16.8%, which is a higher rate than the worldwide prevalence of mental disorders of 13% reported among adolescents.^23^^,^^24^ In our cohort, 7.0% of the participants were diagnosed with ADHD compared to 1–3% in the Danish background population.^23^^,^^24^ One explanation could be that we tested all children in the cohort, including those with mild symptoms or minimal impact from the disease, which differs from those in the background population that gets tested. Another hypothesis is that asthma and ADHD in childhood are associated traits, and the observed parallel increase in incidence may be due to shared environmental risk factors.^5^^,^^24^ In our study, we found that children with asthma in childhood tended to have a double risk of neuropsychiatric disorder at 18 years. The most prevalent neuropsychiatric disorder in our cohort was ADHD, but there was no significant association between ADHD and asthma. In contrast, we observed a significant association between autism and asthma, which has also been demonstrated in some previous studies but not others.^5^^,^^24^, ^25^, ^26^ Interestingly, a study by Qu et al conducted in a US urban cohort of 2580 children showed a significant association between autism and asthma, which diminished after adjusting for maternal factors such as maternal age and weight and child factors such as birth weight and gestational age at birth.^27^ In our study, the association between autism and asthma was still significant after adjusting for maternal age and child gestational age at birth, but we did not adjust for maternal weight or birth weight, which could be possible confounders.

Almost one-fourth of the participants in our cohort reported that they had tried using drugs, which is low compared to the Danish youth population, where nearly 40% have tried cannabinoids, and 10% have tried other types of drugs,^28^ which aligns with what is seen in a study of young Swiss men.^29^ Surprisingly, we found that drug use was lower in adolescents with asthma at age 18 years, where the data was collected anonymously in questionnaires with the involvement of the parents, which could have biassed the answers. We speculate if this could be caused by a disease-specific behaviour, i.e., that having a chronic disease such as asthma causes a lower rate of certain risk behaviours,^30^ especially inhaling drugs as adolescents with asthma compared to adolescents without asthma reported less use of cannabinoids (60.0% vs 70.8%) and laughing gas (6.7% vs 23.6%), whereas there were higher reporting of non-inhalant drugs such as MDMA (26.7% vs 11.1%). Other possible reasons for a lower drug use among adolescents with asthma could also be related to fear of drug interactions with asthma medication, concerns about triggering asthma attacks, and/or increased health awareness/parental supervision in this cohort, where all mothers have a history of asthma. However, previous studies have shown the opposite, ie, living with a chronic disease does not cause less risky behaviour.^31^ In contrast to our findings of less drug use, other studies have shown that adolescents with asthma are more likely to report any drug use compared to healthy adolescents, and it is suggested that this could be a coping mechanism for living with a chronic disease.^3^

We observed that one-fifth reported self-destructive behaviour at some point in their lives. Self-destructive behaviour was associated with asthma in the univariate analyses, and a similar trend was observed in the multivariable models. This corresponds to what has been observed previously, i.e., chronic diseases, such as asthma, may increase the risk of self-harm because of an increased vulnerability from strain and stress and an overrepresentation of depression and anxiety.^32^ However, in our study we did not observe a significant association between either depression or anxiety and asthma although a higher percentage of adolescents with asthma had depression compared to adolescents without asthma (4.3% vs 1.4%, p = 0.11).

We did not find an association between asthma status and alcohol consumption in our cohort. However, an American study found that binge drinking was associated with less asthma control and nonadherence among adolescents with asthma^2^ and another American study showed that adolescents with asthma were more prone to binge drinking as compared to their healthy peers.^3^ Previous studies have found cardiovascular and metabolic side effects related to large alcohol consumption as well as an affected brain development.^33^^,^^34^ In our cohort, two-thirds of the adolescents drank at least 1 time per month, and of these, more than one-fifth drank more than 10 units of alcohol. Of note, only 1% of the participants had never drunk alcohol. However, the alcohol consumption patterns in our adolescents are like that of adolescents from other European countries.^35^ Many initiatives have been initiated to reduce the use of alcohol, and even though consumption is high in our cohort, it is lower compared to previous generations.^36^ A decrease in alcohol consumption has also been observed in countries like Austria and Sweden.^37^^,^^38^ The lack of association with alcohol consumption in our study could be due to the well-controlled analyses, including socioeconomic confounding.

We observed that one-fourth of adolescents with asthma were smokers compared to one-third of adolescents without asthma. There was no association between smoking habits and asthma status in our cohort, which contrasts previous findings in another study showing a significant association between smoking and asthmatic disease, suggesting that adolescents with asthma are more likely to smoke, which could be a coping mechanism for their disease.^3^

We found that the cohort's median daily screen time was 6 h. Previous studies have shown that a high level of time spent on screens per day is associated with adverse health effects such as obesity and impaired cognitive and socioemotional development.^39^ We did not find an association when analysing the correlation between asthma and mean screen time per day. However, daily screen time of at least 8 h per day showed a trend of association with an increased risk of asthma in the univariate analysis, but this was not significant in the multivariable models.

We found that almost one-fourth had a BMI>25, and of those, around 8% were obese. This is consistent with the Danish school-aged children in the background population.^40^ Obesity in childhood and adolescence can negatively affect almost every organ system, thereby causing a broad range of consequences, including hypertension, dyslipidemia, insulin resistance, diabetes, fatty liver disease, as well as psychosocial complications such as low self-esteem and depression.^41^^,^^42^ Many studies have shown an association between asthma and obesity.^43^, ^44^, ^45^ However, we did not observe this in our cohort, which could be due to a lack of power, as the study was not initially designed for this analysis. Nevertheless, we observed a significant association between asthma and obesity among females. This sex-specific association has been observed previously, and it has been hypothesised that sex hormones, especially oestrogen, affect the modulation of Th2 cytokine production, influencing the inflammatory process in the airways and increasing the risk of asthma.^46^^,^^47^ In contrast, other studies have not shown sex differences in the association between asthma and obesity.^48^

### Strengths and limitations

All objective measurements were done by trained research personnel strictly following standard operating procedures. The participants of the COPSAC_2000_ cohort have participated in such examinations repeatedly from birth and are therefore highly competent in performing tests and assessments, which assure a high completion rate. Asthma was diagnosed and monitored at the COPSAC research unit and not by general practitioners or based on parent interviews or questionnaires. The asthma diagnoses were based on objective criteria and treatment, and regular evaluation followed standard operating procedures. Furthermore, all participants were born to mothers with asthma, which may improve symptom recognition. All neuropsychiatric diagnoses were obtained from Danish medical records, encompassing all diagnoses in the national healthcare system. Finally, risk behaviours such as drug use and self-destructive behaviours were assessed in questionnaires, and risk behaviours such as alcohol consumption and smoking were evaluated in the absence of the parents.

A limitation of the study is the external validity because of the high-risk cohort design with all children were born to mothers with asthma, which could have affected our ability to find associations between risk behaviours, neuropsychiatric comorbidity and asthma. Further, all adolescents with asthma in our study had mild to moderate, well-controlled disease, which may have hampered our ability to detect an increased prevalence of risk behaviours and subgroups of neuropsychiatric comorbidity such as ADHD as it is well-know that more severe asthma symptoms and poor control are associated with higher prevalences of neuropsychiatric disorders. In addition, this study is based on a cross-sectional analysis focusing on current asthma at age 18 years, where we labelled children who previously had asthma (i.e., asthma in remission) as healthy controls, which may have introduced a bias towards null findings for association with risk behaviours. Importantly, the birth cohort was not designed to study these associations and null findings in our study concerning risk behaviours could also be due to insufficient power in the analyses and/or residual confounding. All risk behaviours regarding personal integrity were collected; however, underreporting from the participants cannot be excluded and the definition of some risk behaviours such as a high screen time is somewhat arbitrary, whereas other risk behaviours such as self-destructive behaviour and drug use are dichotomized as yes/no to obtain sufficient numbers in the analyses. We collected psychiatric diagnoses from the medical records, including any psychiatric diagnosis, former and present. The diagnoses were divided into 12 sub-groups to simplify the classification and increase the power, which could cause a less precise diagnosis.

## Conclusion

In our cohort at risk of asthma, adolescents with asthma had a higher risk of neuropsychiatric comorbidity and a trend of more self-destructive behaviour. In contrast, drug use was lower compared to adolescents without asthma, whereas other risk behaviours were not associated with asthma. We did not observe an association between obesity and asthma, although sex-stratified analysis did show an association among females. These findings underscore that adolescents with asthma have an increased risk of chronic non-atopic disease and self-harm, which clinicians should be aware of in the regular monitoring and treatment of asthma.

## Abbreviations

ADHD, Attention deficit hyperactivity disorder; ASRS, The Adult ADHD Self-Report Scale; BMI, Body mass index; COPSAC_20000_, Copenhagen Prospective Studies on Asthma in Childhood_2000_; DASS21, Depression, Anxiety, and Stress Scale −21 Items; FeNO, Fractional exhaled nitric oxide; FEV_1_, Forced Expiratory Volume at 1 second.; GA, Gestational age; GLM, Generalised linear model; LSD, Lysergic Acid Diethylamide; MAP, Median arterial pressure; MDMA, 3,4-methylenedioxy-N-methylamphetamine; OCD, obsessive-compulsive disorder; PTSD, post-traumatic stress disorder; sRaw, Specific airway resistance; SDQ, the strengths and difficulties questionnaire

## Ethics statement

The study was conducted following the guiding principles of the Declaration of Helsinki. It was approved by the Local Ethics Committee (COPSAC_2000_: H–B-2008-093, COPSAC_2000_ 18- & 24-years follow-up: H-16039498) and the Danish Data Protection Agency (COPSAC_2000_: 2015-41-3696). Both parents gave written informed consent before enrolment. At the COPSAC_2000_ 18-year visit, the study participants also gave written consent.

## Authors contributions

The guarantor of the study is BC, from conception and design to conduct of the study and acquisition of data, data analysis, and interpretation of data. TME has written the first draft of the manuscript. All co-authors have provided important intellectual input and contributed considerably to the analyses and interpretation of the data. All authors guarantee that the accuracy and integrity of any part of the work have been appropriately investigated and resolved, and all have approved the final version of the manuscript. The corresponding author had full access to the data and had final responsibility for the decision to submit it for publication. No honorarium, grant, or other forms of payment were given to any of the authors to produce this manuscript.

## Submission declaration

The manuscript represents our original work, and it has not been published before.

## Governance

We know and comply with recognized codes of good research practice, including the Danish Code of Conduct for Research Integrity. We comply with national and international rules on the safety and rights of patients and healthy subjects, including Good Clinical Practice (GCP) as defined in the EU's Directive on Good Clinical Practice, the International Conference on Harmonisation's (ICH) Good Clinical Practice guidelines and the Helsinki Declaration. Privacy is important to us, so we follow national and international legislation such as the General Data Protection Regulation (GDPR), the Danish Act on Processing Personal Data, and the practice of the Danish Data Inspectorate.

## Source of funding

The 10.13039/501100003554Lundbeck Foundation (Grant no R16-A1694), the Ministry of Health, Denmark (Grant no 903516), the 10.13039/100007398Danish Council for Strategic Research (Grant no 0603-00280B), and The Capital Region have provided core support to the COPSAC Research Center. Greater Copenhagen Health Science Partners and the Children's Lung Foundation also funded this project.

## Declaration of competing interest

All authors declare no potential, perceived, or actual conflict of interest regarding the content of this manuscript. The funding agencies did not have any role in the design and conduct of the study; collection, management, and interpretation of the data; or preparation, review, or approval of the manuscript. No pharmaceutical company was involved in the study.

## Appendix A Supplementary data

Multimedia component 3 Multimedia component 3

Supplemental Figure 1 : Heatmap showing correlations between risk behaviours included in the study. Supplemental Figure 1

Supplemental Figure 2 . Barplot showing the distribution of psychiatric disorders among adolescents with any disorder in the cohort. Supplemental Figure 2
