# Supplementary material for: Risk behaviours and non-atopic comorbidities of adolescents with asthma
Source: World Allergy Organ J. 2025 Jul 17;18(8):101093. doi: 10.1016/j.waojou.2025.101093 (PMC12296436; doi:10.1016/j.waojou.2025.101093)
Supplement: Multimedia component 1 [file mmc1.pdf]

Supplemental Appendix

METHODS

Data collection followed Good Clinical Practice guidelines. Standard operating procedures were predefined for all study and database registration procedures. We collected data online during the visit. We saved any editing and changes of the data in an audit trail in the database documents. We double-checked the database against source data by an external person.

Non-atopic comorbidity

<u>Neuropsychiatric disorders:</u> The diagnoses were obtained from the medical records based on ICD-10 criteria, including any neuropsychiatric diagnosis, former and present. The diagnoses were grouped into the following groups; ADHD (including the following ICD-10 codes: F.919, F.901, F.900, and F.90), autism spectrum disorder (including the following ICD-10 codes: F.840, F.841, F.8410, F.8412, and F.845), anxiety (including the following ICD-10 codes: F.419, F.41, F.409, F.401, F.410, F.411), depression (including the following ICD-10 codes: F.32, F.321, F.313, F.329, F.339, F.33), eating disorder (including the following ICD-10 codes: F.982 and F.509), mental retardation (including the following ICD-10 codes: F.799, F.79, F.700, F.710, F.849), personality disorder (including the following ICD-10 codes: F.609, F.607, F.606), schizophrenia (including the following ICD-10 code: F.209, F.200), post-traumatic stress disorder (PTSD) (including the following ICD-10 codes: F.439, F.431, F.4302) , obsessive-compulsive disorder (OCD) (including the following ICD-10 codes: F.42, F.429, F.422, F.428, F.959, F.951, F.952), and others (including the following ICD-10 codes: F.941, F.981, F.100).

The strengths and difficulties questionnaire (SDQ) is a tool designed to assess mental health and evaluate an individual's ability to function in daily life. Data from the SDQ questionnaire are presented as the total difficulty/impact scores. We utilised the total difficulty score ranges from 0 to 40, and values above 14 are abnormal [1,2]. The impact score evaluates the impact of difficulties on daily life, is a marker for daily functioning, and varies from 0 to 10. Values of one or above are defined as abnormal [3].

DASS21 is a questionnaire designed to address symptoms of depression, anxiety, and stress. In our study, we used an extended version that also gathered information on drug use and experience with self-destructive behaviour [4].

The Adult ADHD Self-Report Scale (ASRS) is a screening tool for attention and hyperactivity issues. It comprises 18 items, including six main core questions and 12 supplementary questions for preliminary assessment before further evaluation [5].

References

1. Website. Arnfred J, Svendsen K, Rask C, et al. Danish norms for the Strengths and Difficulties Questionnaire. Dan Med J. 2019;66(6). Accessed October 3, 2024. https://pubmed.ncbi.nlm.nih.gov/31256773/

2. Arnfred J, Svendsen K, Rask C, et al. Danish norms for the Strengths and Difficulties Questionnaire.

3. Scheel Rasmussen I, Strandberg-Larsen K, Overbeck G, Wilson P. A critical examination of Danish norms for the Strengths and Difficulties Questionnaire (SDQ). *Nord J Psychiatry*. 2023;77(8):818-823.

4. Henry JD, Crawford JR. The short-form version of the Depression Anxiety Stress Scales (DASS-21): construct validity and normative data in a large non-clinical sample. *Br J Clin Psychol*. 2005;44(Pt 2):227-239.

5. Silverstein MJ, Faraone SV, Alperin S, et al. Validation of the expanded versions of the adult ADHD Self-Report Scale v1.1 symptom checklist and the adult ADHD Investigator Symptom Rating Scale. *J Atten Disord*. 2019;23(10):1101-1110.
