# Supplementary material for: Risk behaviours and non-atopic comorbidities of adolescents with asthma
Source: World Allergy Organ J. 2025 Jul 17;18(8):101093. doi: 10.1016/j.waojou.2025.101093 (PMC12296436; doi:10.1016/j.waojou.2025.101093)

Supplemental Figures

Supplemental Figure 1. Heatmap showing correlations between risk behaviours included in the study.

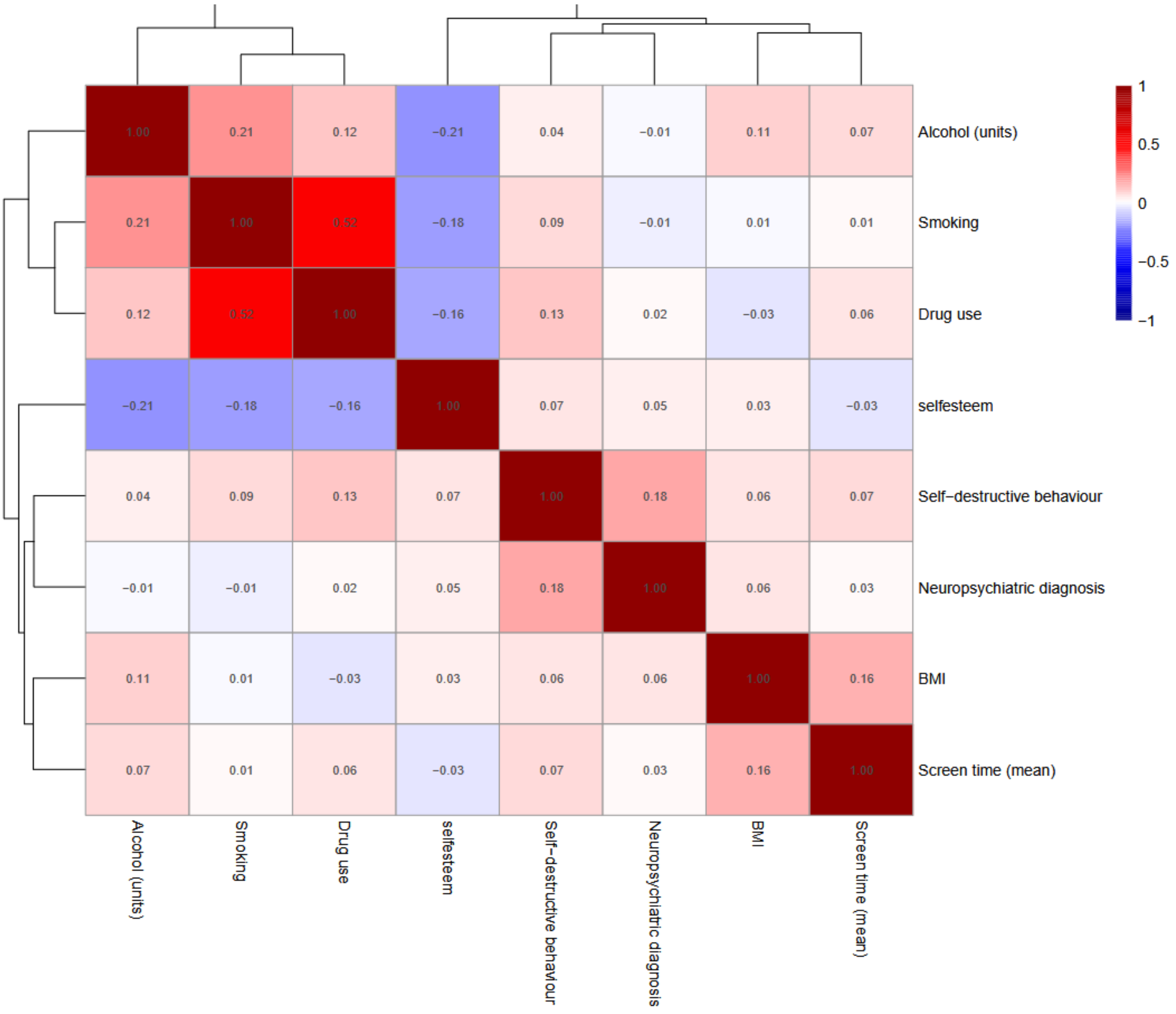

Supplemental Figure 2. Barplot showing the distribution of psychiatric disorders in the cohort through childhood.

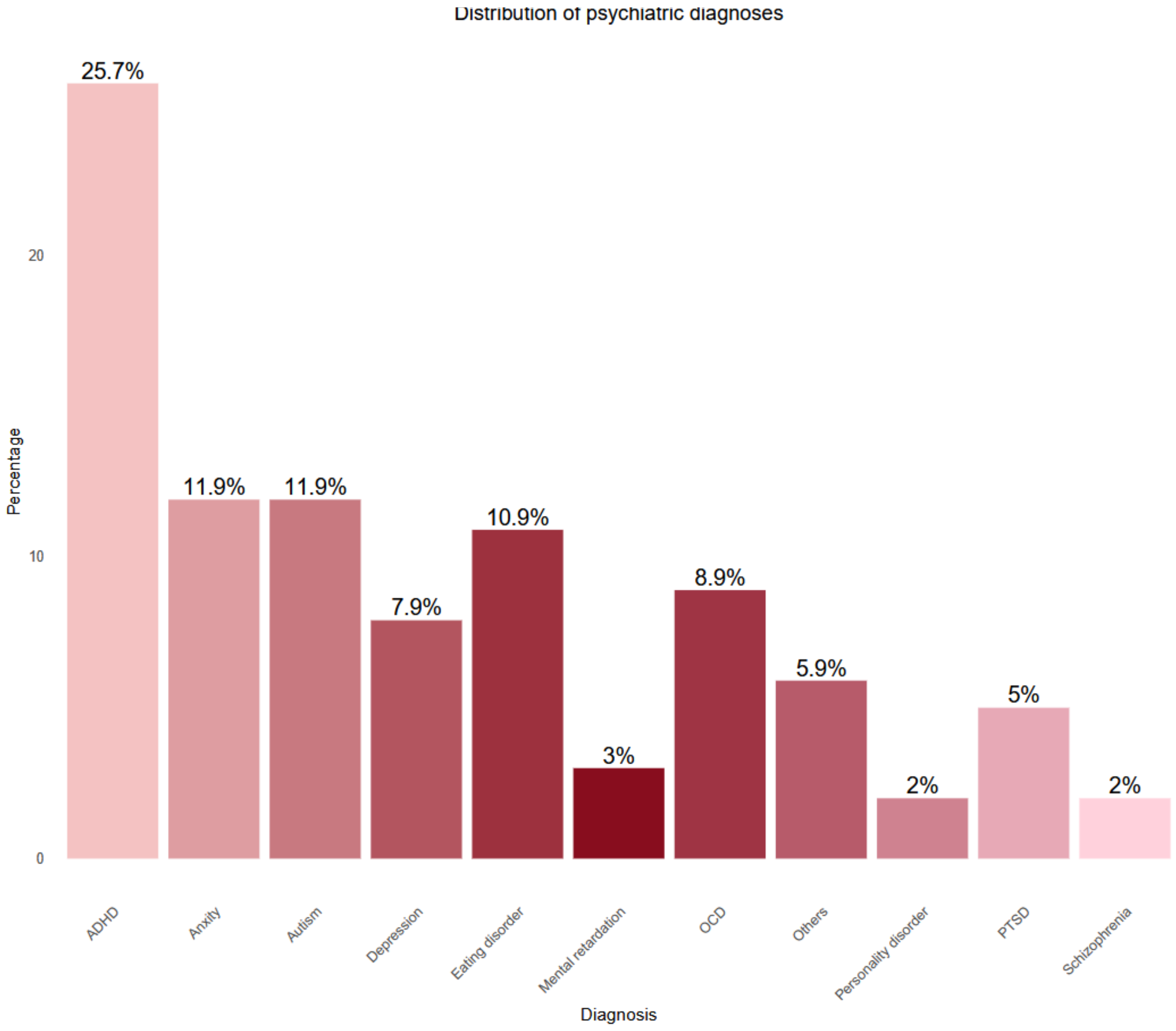

Supplement: Multimedia component 2 [file mmc2.pdf]
