# Supplementary material for: Risk behaviours and non-atopic comorbidities of adolescents with asthma
Source: World Allergy Organ J. 2025 Jul 17;18(8):101093. doi: 10.1016/j.waojou.2025.101093 (PMC12296436; doi:10.1016/j.waojou.2025.101093)
Supplement: Multimedia component 3 [file mmc3.pdf]

Supplemental Table

Table ST1. Asthma associations stratified for gender

Asthma associations stratified for genders	Univariate model females			Univariate model males			[a]Adjusted model females			[a]Adjusted model males			[a] Backward selection females			[a] Backward selection males		
**Predictors**	*OR*	*CI*	*p*	*OR*	*CI*	*p*	*OR*	*CI*	*p*	*OR*	*CI*	*p*	*OR*	*CI*	*p*	*OR*	*CI*	*p*
Binge drinking [b]	1.52	(0.70 – 3.52)	0.30	1.84	(0.78 – 4.85)	0.19	1.31	(0.51 – 3.52)	0.58	2.09	(0.75 – 6.70)	0.18	-	-	-	-	-	-
Current smoking [c]	0.75	(0.25 – 1.96)	0.58	0.91	(0.37 – 2.12)	0.83	0.62	(0.12 – 2.64)	0.54	0.46	(0.10 – 1.82)	0.29	-	-	-	-	-	-
Occasional smoking [c]	0.82	(0.28 – 2.16)	0.70	0.34	(0.08 – 1.10)	0.10	0.82	(0.21 – 2.95)	0.76	0.30	(0.06 – 1.18)	0.11	-	-	-	-	-	-
Drug use	0.53	(0.17 – 1.39)	0.23	0.59	(0.26 – 1.29)	0.20	0.36	(0.07 – 1.47)	0.18	1.18	(0.38 – 3.64)	0.78	0.27	(0.06 – 0.88)	**0.046**	-	-	-
High daily screen time use [d]	1.90	(0.75 – 4.58)	0.16	1.65	(0.77 – 3.42)	0.19	2.19	(0.65 – 7.33)	0.20	1.71	(0.63 – 4.57)	0.28	2.79	(0.92 – 8.38)	0.065	-	-	-
Self-destructive behaviour	2.24	(1.10 – 4.56)	**0.03**	1.31	(0.39 – 3.79)	0.64	1.62	(0.61 – 4.24)	0.32	1.74	(0.47 – 5.85)	0.38	-	-	-	-	-	-
Neuropsychiatric disorder [e]	2.90	(1.26 – 6.56)	**0.01**	1.39	(0.58 – 3.14)	0.44	4.55	(1.40 – 15.78)	**0.01**	1.30	(0.40 – 3.88)	0.65	6.00	(2.03 – 18.74)	**0.001**	-	-	-

Obesity	3.48	(1.18 – 10.33)	**0.02**	1.55	(0.66 – 3.44)	0.30	3.49	(0.81 – 15.75)	0.09	0.42	(0.06 – 2.01)	0.32	-	-	-	-	-	-

Table ST1. Asthma associations stratified for gender

The table shows the association between asthmatics disease at 18 years and predictors. Analyses are made with GLM. Interactions are controlled and included if significant.

[a] Adjusted for sex, GA, mother's age, educational level of the mother, household income, smoking during pregnancy, and breastfeeding. All risk behaviours are included in the multivariable analysis.

[b] Binge drinking refers to alcohol units ≥ 5.

[c] Current smoking refers to smoking weekly, and occasional smoking refers to smoking less than weekly.

[d] High daily screen time use > 8 hours/day.

[e] Neuropsychiatric disorder ever through childhood.
