# Supplementary figures and images for: Risk behaviours and non-atopic comorbidities of adolescents with asthma
Source: World Allergy Organ J. 2025 Jul 17;18(8):101093. doi: 10.1016/j.waojou.2025.101093 (PMC12296436; doi:10.1016/j.waojou.2025.101093)

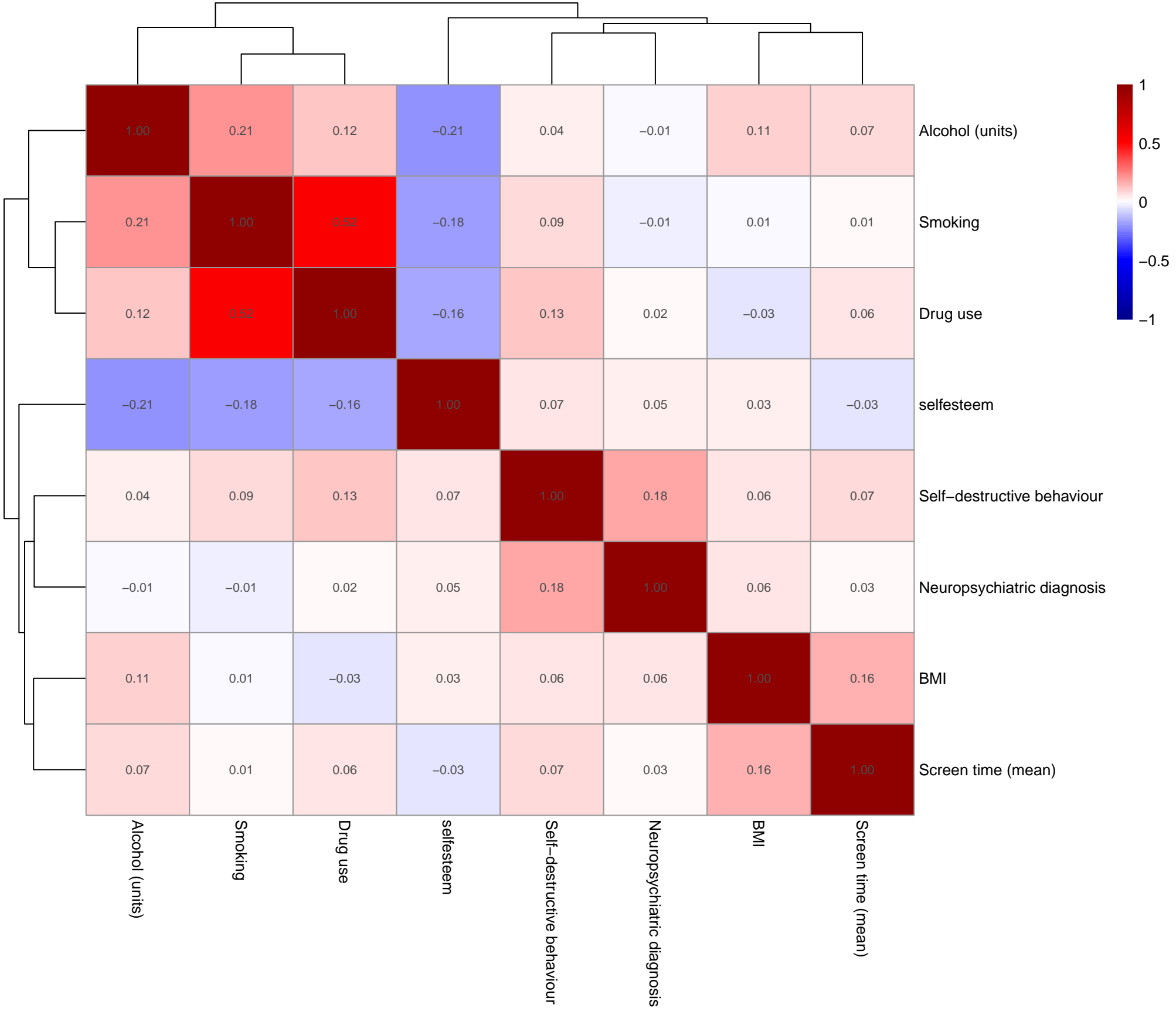

Supplement: Supplemental Figure 1 — : Heatmap showing correlations between risk behaviours included in the study. [file mmc4.pdf]

Distribution of psychiatric diagnoses

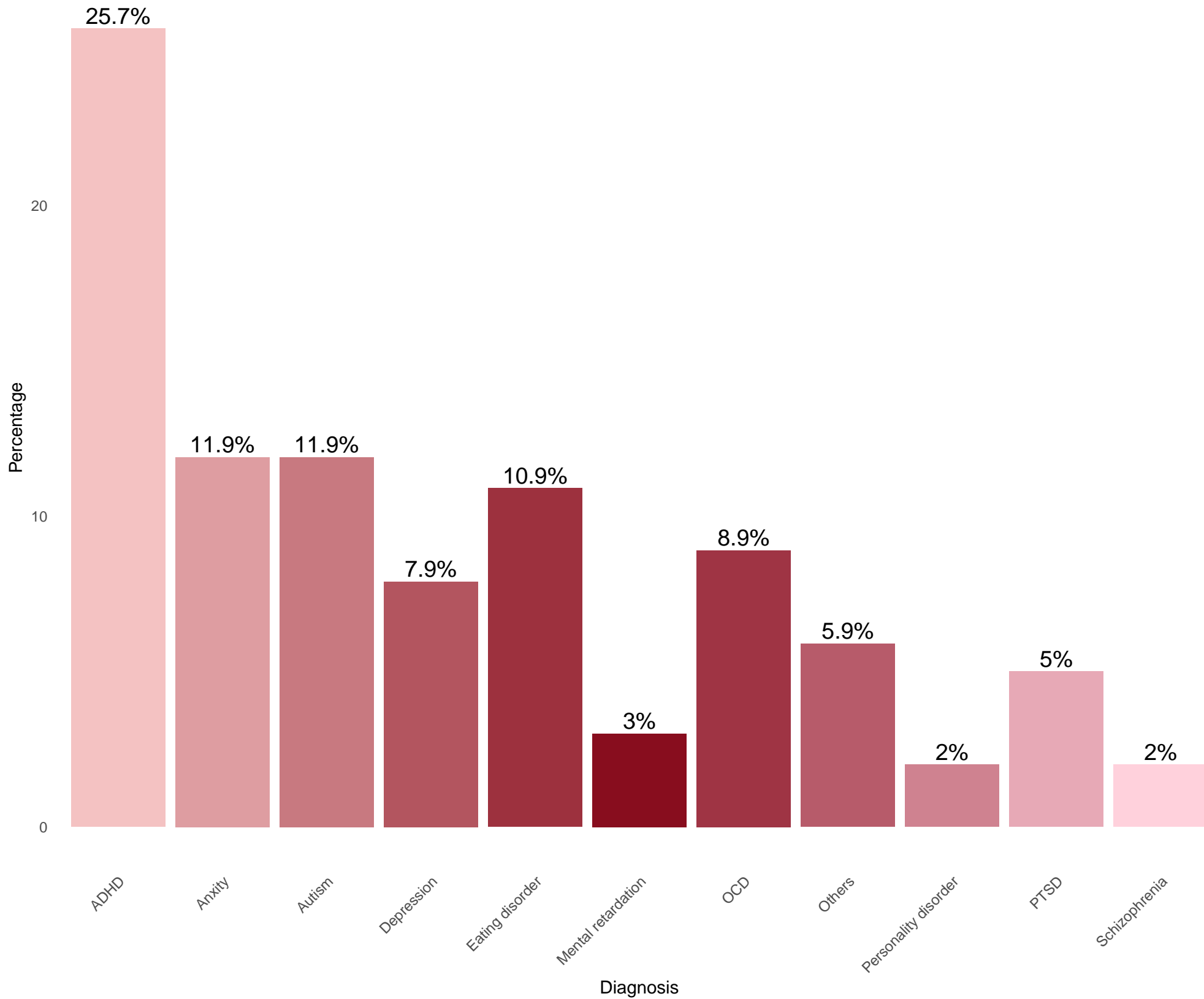

Supplement: Supplemental Figure 2 — . Barplot showing the distribution of psychiatric disorders among adolescents with any disorder in the cohort. [file mmc5.pdf]
